# Socio-demographic determinants of knowledge, attitude and practices towards food safety among Lebanese population during the economic crisis: a cross-sectional study

**DOI:** 10.1186/s12889-022-14808-z

**Published:** 2022-12-13

**Authors:** Rana El Haidari, Fatima Fahes, Fatima Makke, Fatima Nouredine, Kassem Baydoun, Samir Mansour, Abbas Hoballah

**Affiliations:** 1 Department of Research, Islamic Health Society, Baabda, Lebanon; 2 Department of Food Safety, Islamic Health Society, Baabda, Lebanon; 3 Department of Informatics, Islamic Health Society, Baabda, Lebanon; 4 General Director of the Islamic Health Society, Baabda, Lebanon

**Keywords:** Attitude, Economic crisis, Food safety, Knowledge, Lebanon, Practices

## Abstract

**Background:**

Lebanon has been gripped by an economic crisis and the local currency has lost more than 90% of its value; besides, a lack of consistent electricity supply which has contributed to rising concerns about food safety especially among households. This study aimed to assess Lebanese society knowledge, attitude and practice towards food safety during the economic crisis.

**Methods:**

A cross-sectional study was conducted in Lebanon between September 5 and January 30, 2021. Data was collected through an online survey that included information on socio-demographic characteristics, knowledge, attitude and practice of Lebanese consumers towards food safety. Two multivariate regression models were performed on the knowledge and practices as a dependent variable. 95% confidence interval was calculated. All tests were two-sided and statistical significance was set at *p*-value < 0.05.

**Results:**

The majority of participants had good knowledge regarding food safety (74.9%) while more than half of the respondents adopted good preventive practices (62.8%). Multivariate regression of factors associated with good knowledge regarding food safety showed married participants (adjusted OR = 2.1; *p* < 0.0001), who had university degree and above (adjusted OR = 2.0; *p* < 0.0001), and who had income < 1,500,000 LBP (adjusted OR = 1.7; *p* < 0.0001) had a significantly good knowledge score compared to their counterparts. Finally, participants aged more than 35 years old and who had income higher than 1,500,000 LBP (adjusted OR = 1.8; *p* < 0.0001 and adjusted OR = 1.9; *p* = 0.01 respectively) were positively associated to good practice towards food safety.

**Conclusion:**

This study offers useful insights into the knowledge, attitude and practices of Lebanese consumers towards food safety during the economic crisis.

## Background

Since late 2019, Lebanon has plunged into a particularly complex crisis, an economic crisis, followed by the Coronavirus Disease 2019 (COVID-19). As Lebanon, a small middle-income country in the Middle East with a population of six million individuals, suffers from a long history of political and economic instabilities [1]. According to the World Bank, Lebanon is fronting one of the world’s worst economic and financial crises [2]. In 2021, the economy and monetary crisis has reached a critical level which pushed Lebanese families and communities to poverty [3]. Since October 2019, the Lebanese currency collapsed, sliding from 1,500 Lebanese Lira (L.L) to black market value of about 27,000 L.L in late December 2021, and the lira has lost more than 90% of its value on the parallel currency exchange market [4]. Among the downstream effects of this currency devaluation are skyrocketing prices and shortages of basic supplies, including food raw materials, most of which are imported [5].

The unreliable power conditions due to an ongoing fuel crisis leads to a lack of consistent electricity supply, contributing to increasing concerns about food safety especially among households who are unable to keep frozen or chilled foods sufficiently cold to prevent spoilage [6]. Besides, fears over safety and contamination are particularly high for sensitive foods such as dairy, poultry and meat products, whereas canned or dried foods tend to be safer when stored for longer periods of time [7]. Cases of food poisoning have reportedly increased, which is particularly challenging at a time when shortages of fuel and medicine are straining the healthcare system [6, 8]. Besides, the effects of food poisoning can be amplified for individuals who are already malnourished as some professionals fear is the case amidst Lebanon’s current economic crisis [3, 7, 9].

Given the economic burdens that the Lebanese suffer from, it is necessary to evaluate their knowledge, attitude and practices regarding food safety to identify the most prominent gaps that need a preventive and awareness intervention to limit the impact of the economic crisis on their health. The main objective of this study was to assess Lebanese community knowledge, attitude and practice towards food safety during the economic crisis. The second objective was to investigate factors associated with knowledge, attitude and practices regarding food safety during Lebanese economic crisis.

## Methods

### Study design

A cross-sectional study using an online survey was conducted between September 5 and December 30, 2021 among Lebanese consumers across all governorates of Lebanon when the country was under the economic crisis.

### Population

The inclusion criteria were participants aged 18 years old and above, Lebanese and currently residing in Lebanon. Eligible participants were asked to participate in the survey through social media such as WhatsApp groups, Facebook and Instagram. A snowball sampling method was applied using a self-reported online pretested questionnaire formed on Google forms. It was an online survey due to obligatory COVID-19 pandemic lockdown restrictions and limitation of sources to conduct face to face interviews. All participants voluntarily participated in the study and showed to the study objectives.

### Sample size calculation

The sample size was calculated based on the rule of event per variable (EPV) 50 and the sample size formula (*n* = 100 + xi, where x is integer and i represents the number of independent variables in the final model) [10], a minimum sample size of 350 participants must be included in the analysis, a bilateral type 1 error rate of 5%, and a statistical power of 80%, 5 explanatory variables was introduced in the multivariate model.

### Questionnaire

A structured questionnaire was initially developed and designed by a group of 3 environmental engineers with a long experience in food safety based on a thorough review of the available and related literature on the topic, in addition to the latest recommendations and guidelines from the World Health Organization and Libnor [11, 12]. Accordingly, the questionnaire was modified to meet the aim required. The questions were revised to remove the ambiguity and ensure that they were short and clear.

The questionnaire was in Arabic language and consisted of 50 mandatory questions designed as Likert scales questions divided into four different sections:

- 1. Socio-demographic information including age, gender, marital status, education level, professional status, number of persons living in the same house and monthly income. Participants were also asked whether they work in the health field.
- 2. Knowledge section: Four categories with a total of 12 items were designed to measure participant’s knowledge about “keep it clean” (4 items), separation of raw and cooked foods (2 items), keep food at safe temperatures (3 items), and use safe water and safe raw materials (3 items). All the items were answered on a correct/wrong basis and an additional “do not know” option. A correct answer was assigned 1 point and an incorrect/do not know answer was assigned 0 points. The total knowledge score, obtained by the sum of the scores, ranged between 0 and 12. Based on Bloom’s cut off point, participant’s overall knowledge was categorized as good if the score was above 60% (≥ 12 points) and poor if the score was less than 60% (< 12 points) [13].
- 3. Attitude section: Six questions were used to evaluate the fears of the Lebanese community towards food safety during the economic crisis. The items were answered “agree”, “sometimes” and “never” respectively. The answer (agree) was assigned 1 point while answers (sometimes and never) were assigned 0 points.
- 4. Practice section: Nineteen questions were used to assess the practice regarding food safety. Questions were categorized into 4 categories regarding “keep it clean” (5 items), separation of raw and cooked foods (2 items), keep food at safe temperatures (5 items), and use safe water and safe raw materials (7 items). Practice levels were defined as “good” or “poor” based on Bloom’s cut off point. Participants with scores ≥ 60% (≥ 11 points) were classified as having a good practice, while those with scores < 60% (< 11 points) were considered having poor practice [13].

To check for clarity of the questionnaire, a pilot study was conducted which included 15 participants. The Cronbach alpha coefficient value, used to check questionnaire reliability, was observed to be 0.774 for whole questionnaire; and 0.785, 0.723 and 0.775 for the sections knowledge, attitude and practice respectively. The data of pilot study was removed from final analysis. Further modifications were done after feedback retrieval from the participants.

### Data collection

An invitation letter concerning the survey was sent with the link of the questionnaire to groups via social media. The invitation letter includes information describing the survey and asking for a voluntary participation of the Lebanese consumers.

### Statistical analysis

Socio-demographic characteristics of the participants were described using the mean (standard deviation) for continuous variables, and the number (percent) for qualitative variables. Categorical variables were compared in univariate analyses (Pearson chi square test) and the means of continuous variables with the Student’s t-test.

Two multivariate logistic regression models were performed on the knowledge and practices as a dependent variable. Age (≤ 35 years versus > 35 years), marital status (married versus single, separated, divorced or widowed), educational level (secondary school or below versus superior education), employment status (active or not), number of persons living in the same house (≤ 4 persons versus > 4 persons), working in the health field (yes versus no), and income was categorized according to 1,500,000 L.L according to the minimum wage for most employees in the public and private sector in Lebanon (> 1,500,000 L.L versus ≤ 1,500,000 L.L) were assessed as potential predictors in univariate analyses. Variables with a *p*-value ≤ 0.2 in univariate model were eligible for the multivariate model. The collinearity between variables was tested. Variables presenting collinearity were not simultaneously included in the same multivariate model. The variable that has a high variance inflation factor was removed. A stepwise selection approach was then used to select the final multivariate model. 95% confidence interval was calculated.

All tests were two-sided and statistical significance was set at *p*-value < 0.05. The collected data was analyzed with the Statistical Package for Social Sciences software SPSS version 26.

### Ethical aspects

Participants had been informed that their participation was purely on a voluntary basis and their consent was taken prior to starting the questionnaire. The survey did not collect any information exposing the privacy of the participants. The study was conducted according to the guidelines of the Declaration of Helsinki and the Ethics and Research Committee of the Islamic Health Society on August 4, 2021 (reference number 040821–03).

## Results

### Characteristics of the study participants

A total of 555 participants included in the survey among them 81.6% were females. The mean age was 36.7 (SD = 12.0) ranging from 18 to 74 years. The majority of the participants have a university degree (57.1%) and 13.3% of respondents were workers in the health field. The mean number of persons living in the same house was 4.5 (SD = 1.5). The summary of characteristics is shown in Table 1.

**Table 1:** Socio-demographic characteristics of the study population (*n* = 555)

Variable	Frequency	Percentage
**Age, mean (SD)** 36.7 (12.0)		
**Gender**		
Male	102	18.4
Female	453	81.6
**Marital status**		
Married	389	70.1
Single	133	24.0
Divorced	18	3.2
Widow	15	2.7
**Education level**		
Below Primary school	22	4.0
Primary school	109	19.6
High school	107	19.3
University degree and above	317	57.1
**Professional status**		
Active	198	35.7
Retired	8	1.4
Without profession	74	13.3
Housewife	201	36.2
Student	74	13.3
Working in the health field*		
Yes	74	13.3
**Number of persons living in the same house, mean (SD)**	4.5 (1.5)	

Abbreviations: *SD* Standard deviation

^*^Working in the health field: participants who are health care providers (such as nurses, physicians, therapist, pharmacists…)

### Participants’ knowledge of food safety

Out of the 555 participants, the majority 419 (74.9%) had good knowledge. Table 2 describes participants’ answers towards food safety knowledge items. Most of the respondents were aware of the separate raw and cooked foods (90.8%). Knowledge was also good for responses pertaining to the use of safe water and safe raw materials (85.2%) and the category “keep it clean” (84.8%). Poor knowledge was more apparent in response to questions regarding keeping food at safe temperatures (40.9%). The mean total knowledge score was 8.4 (SD = 1.4). There was no statistically significant difference between participants according to gender and working in the health field regard to knowledge (data not shown).

**Table 2:** Responses of study participants to the food safety knowledge questions (*n* = 555)

Knowledge items	Correct		Wrong		Do not know	
	**n**	**%**	**n**	**%**	**n**	**%**
**Category 1. “Keep it clean” (84.8%)**						
Hands must be thoroughly washed and sanitized before preparing food to prevent food contamination (correct)	550	99.1	4	0.7	1	0.2
The facilities of food establishments must be clean, with a certificate evidencing the application of food safety standards (correct)	512	92.3	5	0.9	38	6.8
The use of sterilizers for fruits and vegetables above the permissible levels is safe to eliminate bacteria and microbes (wrong)	101	18.2	390	70.3	64	11.5
Vegetables and fruits can be sterilized by using only drinking water (wrong)	107	19.3	431	77.7	17	3.1
**Category 2. Separate raw and cooked foods (90.8%)**						
Separate equipment such as cutting boards and utensils for raw meat should be used during the preparation process (correct)	521	93.9	13	2.3	21	3.8
Separating vegetables from animal products is important to prevent the transmission of germs (correct)	487	87.7	16	2.9	52	9.4
**Category 3. Keep food at safe temperatures (40.9%)**						
Air humidity is a factor in the transmission of germs (wrong)	454	81.8	24	4.3	77	13.9
Frozen foods are thawed at room temperature (wrong)	372	67.0	156	28.1	27	4.9
Thawed meat can be re-frozen, i.e. re-transferred to the refrigerator (wrong)	36	6.5	502	90.5	17	3.1
**Category 4. Use safe water and safe raw materials (85.2%)**						
The oils used must be healthy and free from any odors and impurities (correct)	546	98.4	1	0.2	8	1.4
Food must be from reliable sources and identified with a label (correct)	528	95.1	5	0.9	22	4.0
The plastic with the number one is only used once (correct)	345	62.2	38	6.8	172	31.0

Correct / wrong in the parentheses denotes the intended (correct) answer. *n* frequency, ***%*** percentage

### The attitude of the Lebanese community towards food safety

Table 3 describes participants’ answers towards food safety during the Lebanese economic crisis. Of all participants, 91.0% feeling reassurance when there is a certificate proving that the facility is subject to control health and permanent audit. Furthermore, 89.7% revealed their fears when buying products of unknown origin; 87.6% exhibited fears towards lack of control over food safety (Table 3).

**Table 3:** The attitude of participants towards food safety

Attitude items	Agree		Sometime		never	
	**n**	**%**	**n**	**%**	**n**	**%**
Reassurance when there is a certificate proving that the facility is subject to health control and permanent audit	505	91.0	40	7.2	10	1.8
Worrying when buying products of unknown origin	498	89.7	38	6.8	19	3.4
Fear of lack of control over food safety	486	87.6	53	9.5	16	2.9
I fear for my family members of food poisoning as a result of the products I buy as offers	363	65.4	88	15.9	104	18.7
Storing food products in large quantities at home for fear of high prices	55	9.9	113	20.4	387	69.7
Buying poor quality food products for fear that they will disappear from the market	46	8.3	46	8.3	463	83.4

*n* frequency, *%* percentage

### The practice of the Lebanese consumers regarding food

Table 4 shows the correct practices regarding the food safety measures. More than half of participants (62.8%) had good practice regarding food safety. The mean total practice score was 15.8 (SD = 2.7). Nearly 72.3% of participants reported taking good preventives practices concerning use of safe water and safe raw materials. Practice was also good for responses pertaining to the separation of raw and cooked foods (70.9%) and the category “keep it clean” (70.1%). Poor practice regarding food safety was more apparent in response to questions regarding keeping food at safe temperatures (59.4%) (Table 4).

**Table 4:** Correct responses of study participants regarding food safety practice questions (*n* = 555)

Practice items		
	Frequency	Percentage
Category 1. “Keep it clean” (49.5%)		
Check the conditions of use and storage of prepackaged food (yes)	462	83.2
Wash cans before consuming them in case there are any contaminants (yes)	416	75.0
Treat changes (color, smell, taste, flavor and shape) of food by increasing flavors and spices (no)	375	67.6
Buying open grains and legumes as they are dry food and exposed to heat during the cooking process (no)	80	14.4
Use sterilizers excessively during the sterilizing of surfaces to eliminate all biological contaminants (no)	43	7.7
Category 2. Separate raw and cooked foods (70.9%)		
Store raw chicken or meat separately from cooked food (yes)	493	88.8
Wash raw meat before cutting or preparing it (no)	295	53.2
Category 3. Keep food at safe temperatures (59.4%)		
Buying foods that need refrigeration in limited quantities to avoid spoilage (yes)	454	81.8
Consume food that has been kept at room temperature for a long time (more than 6 h) (no)	438	78.9
Keep the ice molds to keep cool in case of a power outage to maintain a temperature of about 4 degrees (yes)	353	63.6
Keep leftovers at room temperature until the next meal (such as dinner) (no)	334	60.2
Use the microwave as a way to defrost frozen foods (yes)	72	13.0
Category 4. Use safe water and safe raw materials (72.3%)		
Pay attention to the expiration date before purchasing any product (yes)	530	95.5
I don't buy cans that are dented, swollen or have signs of rust (yes)	485	87.4
Do not consume food after its expiry date (yes)	437	78.7
I buy cleaning and sterilization materials of unknown source (no)	399	71.9
I sift the grains and spices from the licorice and store them again (no)	387	69.7
Recently, I buy the lowest price product even if it is from an unknown source (no)	369	66.5
The moldy part can be removed from vegetables and fruits and the healthy part can be consumed (no)	205	36.9

Yes / No in the parentheses denotes the intended (correct) answer

### Factors associated with good knowledge regarding food safety

Table 5 presents variables significantly associated with knowledge in bivariate and multivariate analyses. The univariate analysis showed an association between married participants (adjusted OR= 1.9; *p* <0.0001), who had university degree (adjusted OR= 2.2; *p* <0.0001), and who had income >1,500,000 LBP (adjusted OR=2.1; *p* <0.0001) and good knowledge score. In the multivariate logistic regression model, married participants (adjusted OR=2.1; *p* <0.0001), who had university degree and above (adjusted OR=2.0; *p* <0.0001), and who had income >1,500,000 LBP (adjusted OR=1.7; *p* <0.0001) had a significantly good knowledge score compared to their counterparts (Table 5).

**Table 5:** Logistic regression analysis between knowledge regarding food safety and socio-demographic variables (*n* = 555)

Bivariate analysis					Multivariate analysis	
	**Poor n(%)**	**Good n(%)**	**Adjusted OR (95% CI)**	***p*-value**	**Adjusted OR (95% CI)**	***p*-value**
**Age (years)**						
≤ 35	52(52.5)	226(50.6)		1.00		
> 35	47(47.5)	221(49.4)	1.0 (0.70–1.67)	0.72		
**Gender**						
Female	83(81.4)	370(81.7)		1.00		
Male	19(18.6)	83(18.3)	0.9 (0.56–1.70)	0.94		
**Marital status**						
Others	43(42.2)	123(27.2)		1.00		1.00
Married	59(57.8)	330(72.8)	1.9(1.25–3.04)	**< 0.0001**	2.1(1.37–3.45)	**< 0.0001**
**Education level**						
High school and below	60(58.8)	178(39.3)		1.00		1.00
University degree	42(41.2)	275(60.7)	2.2(1.42–3.41)	**< 0.0001**	2.0(1.28–3.29)	**< 0.0001**
**Number of persons living in the same house (persons)**						
≤ 4	56(54.9)	222(49.9)		1.00		
> 4	46(45.1)	223(50.1)	1.2 (0.79–1.88)	0.36		
**Working in the Health field**						
No	93(91.2)	388(85.7)		1.00		1.00
Yes	9(8.8)	65(14.3)	0.57(0.83–3.60)	0.14	1.4(0.68–3.13)	0.33
**Income (L.L)**						
≤ 1,500,000	79(77.5)	277(61.6)		1.00		1.00
> 1,500,000	23(22.5)	176(38.9)	2.1 (1.32–3.60)	** < 0.0001**	1.7(1.02–2.91)	**0.04**

Abbreviations: *Adjusted OR* Adjusted odds ratio, *CI* Confidence interval, *LL* Lebanese Lira, *n* frequency, *%* percentage

### Factors associated with good practice regarding food safety

Results of bivariate logistic regression showed that the odds of good practice was 1.5 times higher among adults who are aged over 35 years old (adjusted OR = 1.5; *p* =0.04). Participants with a university degree were 1.6 times more likely to have good practice compared to their counterparts (adjusted OR = 1.6; *p* =0.04). Finally, participants with income >1,500,000 LBP were 2.2 times more likely to have a good practice (adjusted OR = 2.2; *p* <0.0001). In the multivariate logistic regression model, results present that participants aged over 35 years old and who had income higher than 1,500,000 LBP (adjusted OR = 1.8; *p* <0.0001 and adjusted OR = 1.9; *p* =0.01 respectively) were positively associated to good practice towards food safety (Table 6).

**Table 6:** Logistic regression analysis between practice regarding food safety and socio-demographic variables (*n* = 555)

			**Bivariate analysis**		**Multivariate analysis**		
	**Poor n(%)**	**Good n(%)**	**Adjusted OR (95% CI)**	***p*-value**		**Adjusted OR (95% CI)**	***p*-value**
**Age (years)**							
≤ 35	68(59.1)	210(48.7)		1.00			1.00
> 35	47(40.9)	221(51.3)	1.5 (1.00–2.31)	**0.04**	1.8 (1.19–2.93)		**< 0.0001**
**Gender**							
Female	91(79.1)	362(82.3)		1.00			
Male	24(20.9)	78(17.7)	0.8 (0.49–1.36)	0.43			
**Marital status**							
Others	39(33.9)	127(28.9)		1.00			
Married	76(66.1)	313(71.1)	1.2(0.81–1.95)	0.29			
**Education level**							
High school and below	60(52.2)	178(40.5)		1.00			1.00
University degree	55(47.8)	262(59.5)	1.6(1.06–2.42)	**0.02**	0.6(0.42–1.06)		0.08
**Number of persons living in the same house (persons)**							
≤ 4	51(45.1)	227(52.3)		1.00			1.00
> 4	62(54.9)	207(47.7)	0.7 (0.49–1.13)	0.17	0.8(0.52–1.24)		0.33
**Working in the Health field**							
No	106(92.2)	375(85.2)		1.00			1.00
Yes	9(7.8)	65(14.8)	2.0 (0.98–4.23)	0.05	1.8 (0.88–4.01)		0.09
**Income (L.L)**							
≤ 1,500,000	26(22.6)	173(39.3)		1.00			1.00
> 1,500,000	89(77.4)	267(60.7)	2.2 (1.37–3.57)	**< 0.0001**	1.9 (1.1–3.1)		**0.01**

Abbreviations: *Adjusted OR* Adjusted odds ratio, *CI* Confidence interval, *L.L* Lebanese Lira, *n* frequency, *%* percentage

## Discussion

Since the beginning of the economic crisis in Lebanon and the devaluation of the Lebanese currency, Lebanese citizens have started saving their money by exchanging some quality but expensive goods for other cheaper yet less quality goods, buying food offers and buying large quantities fearing its absence. Besides, the lack of knowledge regarding food safety, such as food storage, keeping food at safe temperature and practicing safe buying processes could lead to poisoning and increase the risk of hospital admissions due to food borne pathogens. This study was conducted during the economic crisis that the Lebanese society suffers from.

Our findings that showed good level of knowledge among Lebanese consumers is in line with previous Lebanese studies [14–16]. This could be due to the fact that, most participants were educated and working in the health-related majors and this can lead to high awareness regarding food safety. Conversely, several recent studies conducted in the Middle East showed a low to moderate level of knowledge regarding food safety such as in Saudi Arabia [17, 18], Jordan [19] and Egypt [20]. Marriage, higher education and higher income were significant predictors of good knowledge score. Married consumers were more conscious about food safety than their unmarried counterparts because they bear the responsibility of their families; consequently, they have awareness in the purchase process in terms of providing what is best for their families. These results have been discussed by several researchers [17, 21–23]. Furthermore, we found that higher educated participants had higher food safety knowledge score; this agree with that of Hassan and Dimassi (2014), Liu and Niyongira (2017), and Alhashim (2022), who reported that educated consumers pay more attention to food safety as compared to consumers with low education levels [18, 24, 25].

Recently, several Lebanese studies showed the weakness in food security and the fear of Lebanese consumers from the impact of the economic crisis on the safety of their food, and this is consistent with the results of our study [7, 26, 27]. Kharoubi et al. highlight the alarming trends in food insecurity prevalence in Lebanon where was 3.1% of the Lebanese population in 2015–2017, then was projected to reach up to 20% and 24% of the population by 2020 and 2022, respectively [28]. These results highlight the importance of intensifying the field of food safety control on Lebanese food establishments.

Aligning with findings from a previous study among residents of Lebanon during the COVID-19 pandemic [16], the present study reported that 62.8% of the participants have good level of practice towards food safety; which is higher to the score (71.3%) reported by Hassan et al. (2018) among domestic food handlers in Lebanon, and that (53.6%) reported by Hassan and Dimassi (2014) among Lebanese university students, knowing the aforementioned two studies did not use exactly the same questions as in the present study [15, 24]. Besides, good practice of food safety was significantly influenced by higher age and higher monthly income. In light of the economic crisis, the monthly income plays a key role in maintaining sound applications, such as purchasing well-known goods and permanently securing electricity to keep food at an ideal temperature. These results reflect the situation of participants when the study was being held during the economic crisis period where the exchange rate was fluctuating and socio-economic status of people in Lebanon was changing from week to week. For this reason, the results must be interpreted with caution.

Several limitations should be considered. Due to the cross-sectional design, this study does not allow us to found a causal association. Further studies that incorporate a longitudinal design would offer causal interference for consumers’ knowledge, attitude and practice towards food safety issues. No validated tool for the assessment of the knowledge, attitude and practices of food safety was available. Some items were formulated from the World Health Organization and Libnor for the assessment of knowledge, attitude, and practice toward food safety (11, 12). Because of the lockdown, we did not design the sample to statistically represent the Lebanese population and make rigid extrapolations. We gathered data through online survey links. However, a response barrier for some participants might have been created due to that unavailability of the internet to everyone in Lebanon and the fact that the older generation is computer illiterate. Furthermore, respondents’ self-reported responses may cause information and reporting bias. However, the study has several strengths. The findings may provide evidence for policymakers and public health experts to plan an evidence-based involvement across Lebanon to improve consumers’ knowledge, attitudes and practices on food safety and to prevent foodborne illness. Moreover, a higher sample size distributed across all Lebanese governorates represents an additional potency in this study.

## Conclusion

This preliminary study revealed an appropriate level of knowledge and good practices towards food safety during the economic crisis among Lebanese consumers; noting some gaps on food safety practice that can affect their health. Several socio-demographic factors were associated with participants’ knowledge (marital status, education level and income) and practice (age and income) towards the safety of food. However, given the associated public health risk that can result from inadequate food safety, there is a necessity for targeted national strategies and intervention programs, such as educating about foodborne pathogens and their adverse impact, food safety training and awareness campaigns to improve consumers’ knowledge concerning food safety.

## Data Availability

The datasets used and/or analyzed during the current study are available from the corresponding author on reasonable request.

## Abbreviations

Adjusted OR: Adjusted odds ratio
COVID-19: Coronavirus disease 2019
CI: Confidence interval
SD: Standard deviation
LL: Lebanese Lira
